# Self-Reported Insomnia and Poor Sleep Quality Are Associated with Self-Reported Cognitive Changes in Older Adults

**DOI:** 10.3390/clockssleep7040056

**Published:** 2025-10-02

**Authors:** Julia Glueck, Celina Pluim McDowell, Yakeel T. Quiroz, Alice Cronin-Golomb, Jeanne F. Duffy

**Affiliations:** 1 Department of Psychological and Brain Sciences, Boston University, Boston, MA 02215, USA; jglueck@bu.edu (J.G.); cpluim@bu.edu (C.P.M.); yquiroz@bu.edu (Y.T.Q.); alicecg@bu.edu (A.C.-G.); 2 Division of Sleep and Circadian Disorders, Department of Medicine, Brigham and Women’s Hospital, Boston, MA 02115, USA; 3 Department of Psychiatry, Massachusetts General Hospital, Harvard Medical School, Boston, MA 02114, USA; 4 Department of Neurology, Massachusetts General Hospital, Harvard Medical School, Boston, MA 02114, USA; 5 Division of Sleep Medicine, Harvard Medical School, Boston, MA 02115, USA

**Keywords:** mild cognitive impairment, cognitive function, aging, sleep disturbance

## Abstract

Older adults are vulnerable to changes in sleep with age. Poor sleep quality is associated with self-reported cognitive changes, which can occur before the onset of objective cognitive decline associated with Mild Cognitive Impairment and Alzheimer’s disease. The objective of this study was to examine associations between self-reported sleep complaints, objective sleep quality, and self-reported cognitive changes and their relations to symptoms of depression and anxiety in a group of community-dwelling older adults. Adults aged ≥ 50 without dementia (*n* = 45) were recruited and completed 1–2 weeks of rest-activity monitoring using a wrist-worn device, underwent a test of global cognitive functioning (Mini-Mental State Examination; MMSE), and completed questionnaires assessing insomnia (Insomnia Severity Index; ISI), subjective sleep quality (Pittsburgh Sleep Quality Index; PSQI), self-reported cognitive changes (Cognitive Function Instrument; CFI), and symptoms of depression and anxiety (Beck Depression Inventory-II; BDI-II and Generalized Anxiety Disorder 7-item assessment; GAD-7). Pearson partial correlations assessed relations among subjective and objective sleep quality, insomnia ratings, CFI ratings, and global cognition, while controlling for BDI-II and GAD-7 ratings. Exploratory analyses examined the correlation between PSQI component scores and CFI ratings and global cognition. Greater ISI (*r* = 0.50, *p* ≤ 0.001) ratings significantly correlated with higher CFI scores. PSQI total ratings and actigraphy-based measures (*n* = 41) did not significantly correlate with CFI scores. Exploratory PSQI subscale analyses revealed that worse subjective sleep quality (*r* = 0.31, *p* = 0.048), shorter sleep duration (*r* = 0.32, *p* = 0.04), and greater use of sleep medications (*r* = 0.31, *p* = 0.048) correlated with higher CFI scores. Poorer sleep quality due to less time spent asleep, fragmented or disturbed sleep, and requiring medications to sleep, may be associated with greater memory concerns. Alternatively, worries about cognition may deleteriously affect sleep. Subjective measures of sleep quality may be useful to identify older adults at increased risk of cognitive decline.

## 1. Introduction

Sleep disruption and insomnia complaints are common features of aging, and typically include earlier bed times, sleep fragmentation, less time spent in slow-wave sleep, and waking earlier than desired [1,2]. Insomnia Disorder is defined by the American Psychiatric Association as difficulty initiating sleep, maintaining sleep, and/or early morning awakenings that cause distress and/or functional impairment [3]. Insomnia Disorder impacts approximately 10% of adults in the general population, although an additional 25–30% of adults may experience occasional sub-diagnostic insomnia symptoms [4], and as many as 50% of older adults endorse problems initiating or maintaining sleep [5]. Sleep–wake disturbances are also seen in the early stages of neurodegenerative disorders, including Alzheimer’s disease (AD), but it is yet unclear if sleep disturbances are a symptom or a cause of progression of AD [2,6].

In addition to sleep changes, older adults may report subjective changes in cognition that may or may not be accompanied by objective evidence of decline on neuropsychological testing. Self-reported cognitive changes without objective evidence may indicate an early stage of decline related to Alzheimer’s disease, and have been associated with an increased likelihood of future dementia and AD-related biomarkers [7,8,9,10]. Therefore, self-reported cognitive changes represent an important research target for understanding the progression of cognitive decline associated with aging and neurodegeneration.

Research suggests a relation between poor sleep quality and risk of cognitive decline in older adults. In several studies, worse sleep quality has been associated with poorer subjective cognitive performance in middle-aged and older adults [11,12,13,14]. Some studies suggest that these relations are associated with and possibly mediated by symptoms of depression and anxiety, so it is important to consider this when evaluating sleep quality and subjective cognitive performance [15,16,17,18]. Insomnia Disorder in older adults has also been shown to correlate with worse memory performance on neuropsychological tests [4]. Measures of insomnia used across these studies varied widely, ranging from a single question to a clinical diagnosis meeting DSM criteria [14], and many were limited to self-report measures of insomnia or sleep quality and did not include objective measures such as actigraphy or polysomnography. Notably, self-report measures of sleep quality in healthy adults do not necessarily correlate with objective sleep data, suggesting that some people may not have an accurate perception of their own sleep [19,20]. Therefore, it is important to include objective sleep measures when examining the relation between sleep and cognition.

The current study examined associations between self-reported cognitive changes, global cognitive function, and sleep disturbances in older adults without dementia using self-report measures and wrist actigraphy. The aims were to (1) examine associations of subjective sleep quality ratings and subjective insomnia ratings with subjective cognitive change ratings and global cognitive functioning, and (2) examine associations among objective sleep, self-reported cognitive changes, and global cognition. We hypothesized that worse subjective insomnia, worse subjective sleep quality, and objective measures of sleep disturbance would correlate with greater scores on a measure of self-reported cognitive changes, as well as with worse global cognitive function.

## 2. Results

### 2.1. Study Sample

Characteristics of the study sample are summarized in Table 1. On average, participants were 64.2 years old with 16.1 years of education and intact cognitive functioning. The racial and ethnic composition of the group was Asian (*n* = 3), Black or African American (*n* = 7), Hispanic/Latino (*n* = 4), Non-Hispanic White (*n* = 32), and more than one race (*n* = 3). Participants endorsed average scores of 2.1 (SD = 2.4) on the CFI, indicating mild subjective cognitive changes; 7.0 (SD = 5.9) on the ISI, indicating subthreshold insomnia; and 6.0 (SD = 3.9) on the PSQI, indicating disturbed sleep. Participants endorsed average BDI-II ratings of 4.2 (SD = 4.9), and average GAD-7 ratings of 1.8 (SD = 2.3).

### 2.2. Association Between Scores on Cognitive Measures and Subjective Sleep Complaints

**Figure 1:**
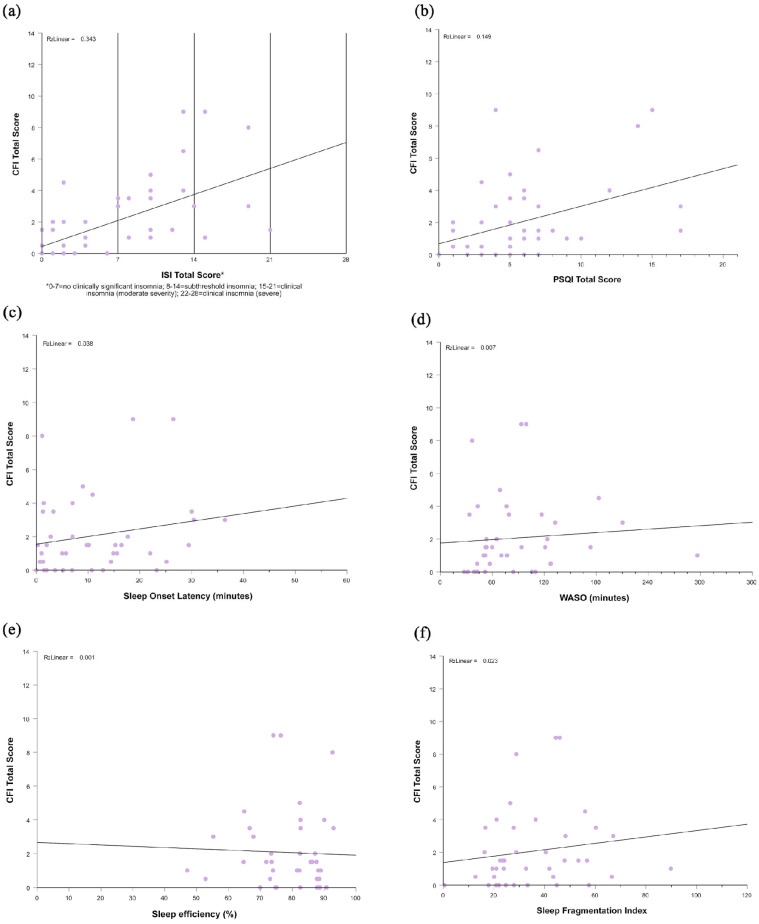
Correlations between scores on subjective and objective sleep data. (**a**) CFI and ISI total scores; (**b**) CFI and PSQI total scores; (**c**) CFI scores and sleep onset latency as measured by actigraphy; (**d**) CFI scores and WASO as measured by actigraphy; (**e**) CFI scores and sleep efficiency as measured by actigraphy; (**f**) CFI scores and sleep fragmentation index as measured by actigraphy.

Correlations between age, education, BDI-II scores, GAD-7 scores, and cognitive measures were assessed to identify potential covariates. BDI-II scores (r = 0.62, *p* ≤ 0.001) and GAD-7 scores (r = 0.31, *p* = 0.04) were significantly correlated with CFI scores, but not with MMSE scores (p’s > 0.3; Table 2). Age and education did not significantly correlate with CFI or MMSE scores (p’s > 0.06; Table 2); however, because age is known to be associated with changes in sleep and cognitive function, we included this as a covariate in our analyses. When controlling for age, BDI-II scores, and GAD-7 scores in the subsequent Pearson partial correlations, we found that higher ISI ratings (r = 0.50, *p* = <0.001) were significantly correlated with higher CFI ratings (Table 2, Figure 1a). PSQI total score was not correlated with CFI ratings (r = 0.28, *p* = 0.07) (Table 2, Figure 1b). Neither ISI nor PSQI ratings were correlated with MMSE ratings (p’s > 0.10; Table 2).

In an exploratory analysis of the seven PSQI subscales, we found that worse subjective sleep quality (r = 0.31, *p* = 0.048), shorter sleep duration (r = 0.32, *p* = 0.04), and greater use of sleep medications (r = 0.31, *p* = 0.048) were significantly correlated with higher CFI scores. None of the PSQI subscales were correlated with MMSE score (p’s > 0.09; Table 2).

### 2.3. Association Between Scores on Cognitive Measures and Objective Sleep Data

Actigraphy data were available from 41 participants, with an average of 8.5 days of data from each participant (SD = 2.7, range = 6–15). When controlling for age, BDI-II scores, and GAD-7 scores, none of the actigraphy measures, including SOL, TST, sleep efficiency, WASO, number of awakenings, and sleep fragmentation index, correlated with CFI scores (p’s > 0.20; Table 3, Figure 1c–f) or MMSE scores (p’s > 0.10; Table 3).

## 3. Discussion

We examined associations between subjective cognitive changes, global cognitive functioning, subjective insomnia and sleep quality, and objective sleep disturbance. We found that when controlling for anxiety and depression ratings, greater subjective insomnia symptoms and worse subjective sleep quality ratings on PSQI subscales were both associated with higher CFI scores, reflecting greater subjective perception of cognitive changes. In contrast, objective sleep measures assessed with actigraphy were not significantly associated with higher CFI scores in our sample. Neither subjective nor objective sleep measures were associated with global cognition as measured by the MMSE.

The finding that poorer subjective sleep quality and greater subjective insomnia symptoms were associated with greater CFI scores is consistent with previous studies in middle-aged and older adults [11,12,13]. Some of those studies assessed subjective cognitive changes using only one or two questions, so our analyses add to the current research by employing a more comprehensive measure of subjective cognitive changes. Additionally, our exploratory analysis of the PSQI component scores demonstrates a relation between higher CFI scores and three of the seven PSQI subscales: sleep quality, sleep duration, and the use of sleep medication. These results suggest that older adults may endorse problems such as poorer sleep quality, shorter sleep duration, and an increased reliance on sleep medications early on during the course of cognitive changes with aging, and specific sleep difficulties reported by patients could signal their likelihood of experiencing concurrent or future cognitive changes. Thus, easy-to-administer subjective measures assessing sleep quality and/or insomnia symptoms may help to identify older adults who may be experiencing subjective cognitive changes that warrant follow-up objective assessment.

Though self-reported sleep concerns correlated with greater CFI scores, we found that actigraphic sleep data were not correlated with either CFI scores or global cognition. Our results are somewhat consistent with a study by Jiang et al., who found that subjective insomnia (measured by the ISI) correlated with self-reported cognitive changes (measured by the Subjective Cognitive Decline Questionnaire 9 (SCD-Q)), but not with actigraphic measures of sleep quality; however, that study found no correlation between self-reported cognitive changes and PSQI scores [21]. The authors observed through a mediation effect analysis that depression (measured by the Geriatric Depression Scale) appeared to mediate the association between subjective insomnia and self-reported cognitive decline [21]. Other prior studies provide mixed data on the relations between self-reported cognitive changes and actigraphy. Some studies have found that older adults reporting subjective cognitive changes (measured by the SCD-Q) showed shorter sleep durations, more wake during sleep, and lower sleep efficiency on actigraphy compared to adults without subjective cognitive decline [22,23]. By contrast, other studies have found an association between greater subjective cognitive changes and better-quality sleep, including longer sleep duration, fewer awakenings and fewer minutes of wake during sleep, lower sleep fragmentation, greater sleep efficiency, and less time in bed as assessed using polysomnography and actigraphy [24,25]. The authors of those studies hypothesized that better sleep quality may be a compensatory mechanism for greater daytime cognitive effort in people experiencing subjective cognitive decline; alternatively, individuals with poorer sleep may have habituated to their cognitive issues and may experience them as less serious, thus reporting fewer subjective cognitive changes [24,25].

There are several reasons why our findings are inconsistent with these studies. First, our participants were cognitively intact with low average CFI ratings, whereas prior studies included samples with more significant subjective cognitive concerns. As the above studies suggest, it is possible that as more subjective cognitive concerns are reported, sleep quality appears to improve (e.g., greater TST or less WASO) as a compensatory mechanism for cognitive changes, though this needs to be investigated in longitudinal studies. Additionally, there is no one standard measure of subjective cognitive concerns, and differences in the instruments used to measure this could contribute to the discrepancy between studies. Future studies investigating subjective cognitive and sleep changes may benefit from using the same measures so that results can be compared between different studies. It is also possible that both disrupted sleep and excessively long sleep are correlated with a perception of cognitive changes. Previous studies have found associations between self-reported excessively short or long sleep and lower objective cognitive performance in older adults [26], and sleep disruption and sleep restriction are correlated with objective cognitive changes in healthy younger adults [27,28], but more research is needed to determine if these associations hold for self-reported cognitive changes as well.

In the current study, several possible reasons could explain the discrepancy in the relations between self-reported sleep changes versus objective sleep measures and subjective cognitive concerns. One possibility is that the older adults in our study may feel tired or sleepy and attribute this to shorter or disrupted sleep, when it could instead be due to other causes. Prior research suggests that fatigue is a common complaint in older adults, with one meta-analysis suggesting a prevalence of 42.6% [29]; older adults may attribute general fatigue to sleep changes, when it could instead be a normal feature of aging or related to other health issues. Alternatively, older adults may be experiencing greater anxiety or worries about decline in multiple areas, leading to poorer ratings on both subjective cognitive measures and subjective sleep quality measures, without concurrent evidence of objective changes in either area. This possibility is supported by a study by Sun et al., which found that both worries (as measured by the worry scale of the Old Adult Self Report (OSAR)) and anxiety/depression (measured by the anxious/depressed subscale of the OSAR) serve as distinct mediators of the relationship between subjective sleep quality (measured by the PSQI) and subjective cognitive concerns (measured by the memory/cognition problems subscale of the OSAR) [16]. A third possible explanation for our findings is that subjective sleep concerns might represent an early phase preceding objective sleep changes, in a similar way that subjective cognitive concerns can precede objective cognitive decline; or that the actigraphy measures used in our study are not sensitive enough to pick up on very early sleep changes, and older adults may be sensing changes that are occurring but not yet visible on actigraphy. Because actigraphy relies on wrist movement data to extrapolate sleep patterns [30], very minor changes in brain activity during sleep may not be detected by the actigraphy monitor. Therefore, the use of more sensitive sleep measures, such as polysomnography and electroencephalography, in future studies could provide insight on whether early sleep changes are actually occurring, or whether subjective sleep changes reported by older adults are instead due to other reasons, such as a lack of insight, negative self-perception of aging, or lack of knowledge of normal sleep changes related to aging.

Because of the cross-sectional design of this study, it is not possible to determine whether subjective sleep disturbances may cause a perception of cognitive changes, or whether the mechanisms that lead to subjective cognitive concerns also lead to subjective sleep problems. It is also possible that worries about cognitive decline may negatively impact sleep quality, or as noted above, that older adults may experience greater worries about health changes in general, driving the experiences of both subjective sleep and subjective cognitive changes. Longitudinal research is needed to explore these possible patterns and their directionality further. While some longitudinal studies have examined the relations between subjective and objective cognitive changes and objective sleep changes [22,31], fewer studies have incorporated measures of subjective sleep changes; therefore, incorporating easy-to-administer questionnaires such as the PQSI and ISI, as well as measures examining general worries about health changes/decline, in longitudinal cohort studies could provide more insight on the relations between perceived changes in sleep and cognition. If disrupted sleep leads to a perception of cognitive decline, then addressing the causes of disrupted sleep could be important clinically to reduce worries about cognition, and potentially to reduce the chance of decline that may later progress to cognitive impairment or dementia. Possible interventions could include educating individuals about sleep hygiene (behavioral and environmental changes conducive to better sleep, such as adhering to a regular bedtime and waketime, limiting light and noise sources in the bedroom), conducting systematic screening for sleep disorders such as obstructive sleep apnea that are highly prevalent among older individuals, and/or evaluating for and addressing medical or psychological issues leading to difficulty falling asleep, frequent nighttime awakenings, or medication reliance (e.g., unmanaged pain, anxious thoughts, medication side effects). If subjective cognitive concerns precede the onset of sleep problems, then self-reporting of cognitive changes could be a flag to clinicians that there may be a risk of sleep problems, warranting further monitoring and follow-up. Likewise, addressing subjective cognitive concerns (by providing psychoeducation about cognitive changes associated with normal aging, and/or by implementing strategies to aid cognitive functioning in daily life) may also reduce the experience of subjective sleep problems.

We found no correlation in our sample between subjective or objective sleep quality and global cognition. Another study enrolling community-dwelling older adults found correlations between poorer cognitive performance on the MoCA-Survey Adaptation (MoCA-SA) [32] and scores from several actigraphic measures of sleep quality, including a greater number of awakenings and more minutes of wake during sleep, higher sleep fragmentation, and lower sleep efficiency [31]. Our study’s results may be discrepant because our sample was largely cognitively intact. It is possible that objective sleep quality decline occurs later on in the process of age-related cognitive changes after objective cognitive impairment is present, rather than earlier when only subjective changes are reported. Future studies should include more heterogeneous samples, including those with more cognitive decline, to better examine these associations.

This study was subject to limitations. Our sample was highly educated, with participants having a college education on average. Future studies that include participants from more diverse educational backgrounds will help generalize findings regarding associations of sleep measures with SCCs. Our sample was also majority Non-Hispanic White, and more work is needed to determine if the associations between cognitive changes and sleep apply to other racial and ethnic groups. Future research should examine a larger sample that includes more diverse participants, including those from other racial, ethnic, and socioeconomic backgrounds, allowing for greater generalizability of results and possible subgroup analyses to explore differences in sleep and subjective cognitive changes among these groups. Additionally, future research would benefit from the inclusion of participants with greater subjective cognitive concerns than those in our sample, as well as participants reporting a wider range of mood symptoms. Finally, our study is cross-sectional, preventing us from determining whether subjective cognitive changes or sleep disturbance arise first, or together, in cognitively intact older adults.

## 4. Materials and Methods

### 4.1. Participants

Individuals aged 50 and older living in the community were recruited from the greater Boston area. The inclusion criterion was a Telephone Interview for Cognitive Status (TICS) [33] score > 27. Exclusion criteria comprised a diagnosis of dementia (self-reported) or neurodegenerative disease; history of stroke, coma, major head injury, brain surgery, or cancer of the head or nervous system; untreated major depression or anxiety; history of psychosis; current alcohol or substance abuse; use of beta blockers; use of melatonin/melatonin agonists within the past month; and a history of overnight shift work in the past three years. Participants completed an interview including demographic information, medical history, medications, anxiety and mood symptoms, and sleep habits, as well as a short assessment of global cognition. The initial sample in this study included 53 participants. Two participants were excluded because they met the criteria for mild cognitive impairment. For participants missing data pertinent to the analyses (i.e., missing items from subjective sleep or cognitive measures), listwise deletion was implemented. The final sample comprised 45 participants. Study procedures were approved by the Mass General Brigham Institutional Review Board. Participants gave written informed consent before study initiation and were compensated for their time.

### 4.2. Sleep Measures

#### 4.2.1. Actigraphy

CamNtech MotionWatch 8 wrist actigraphy monitors were used to track activity, sleep, and ambient light exposure (CamNtech Inc., Boerne, TX, USA). Participants were instructed to wear the monitor on their non-dominant wrist at all times (except when swimming or bathing) for 1–2 weeks and keep a sleep–wake diary during this time. Data were processed using CamNtech MotionWare 1.3.33 software (CamNtech Inc., Boerne, TX, USA). Bed and wake times were determined using a combination of actigraphy data and sleep diaries; software-determined bed and wake times were cross-referenced with sleep diary entries, and if a significant discrepancy existed, sleep times noted in the sleep diaries were used. Outcome measures included total sleep time (actual amount of time asleep during the main sleep episode, not including daytime naps; TST), sleep efficiency (percent of time in bed spent asleep), sleep onset latency (number of minutes from getting into bed to fall asleep; SOL), wake after sleep onset (total amount of time spent awake after initiating sleep; WASO), number of awakenings (number of awakenings during the sleep period; NA), and sleep fragmentation index (ratio of NA to TST in minutes; SFI) [30,34]. All actigraphy measures were derived from MotionWare. Wrist actigraphy data were available from 41 participants.

#### 4.2.2. Insomnia Severity Index

The Insomnia Severity Index (ISI) is a self-report questionnaire evaluating sleep problems (sleep onset, sleep maintenance, early morning awakening, satisfaction with current sleep pattern, impact on daily functioning, level of impairment due to sleep problems, and level of distress due to sleep problems) [35,36]. Respondents rate sleep problems across seven items on a five-point scale from 0 (“not at all”) to 4 (“extremely”) during the last two weeks. Total scores range from 0 to 28, with higher scores indicating greater insomnia severity (scores 15 or greater indicate clinical insomnia) [35]. The scale has been validated in healthy adults and in patients with insomnia, including older adults ages 55–84 with primary insomnia [35,36,37].

#### 4.2.3. Pittsburgh Sleep Quality Index

The Pittsburgh Sleep Quality Index (PSQI) is a 19-item self-report questionnaire to rate overall sleep quality experienced during the past month using a combination of 4-point scale questions and open-ended responses [36]. The questionnaire yields an overall score and 7 component scores representing subjective sleep quality, sleep latency, sleep duration, sleep efficiency, sleep disturbance, use of sleep medication, and daytime dysfunction. The component scores range from 0 (no difficulty) to 3 (severe difficulty), and the total score ranges from 0 to 21, with a higher score indicating worse sleep quality and scores greater than 5 indicating disturbed sleep. The PSQI has been validated in numerous community and clinical populations, including healthy older adults [38,39,40].

### 4.3. Cognitive Measures

#### 4.3.1. Cognitive Function Instrument

The Cognitive Function Instrument (CFI) is a 14-item self-report questionnaire assessing self-reported cognitive changes. Questions ask about difficulties with memory, functional activities, and social activities [41] now compared to one year ago. Response options include “yes,” (1 point), “no” (0 points), “maybe” (0.5 points), and “not applicable” [41]. Individual responses are summed into an overall score with higher scores indicating greater subjective cognitive impairment (range: 0–14) [41].

#### 4.3.2. Mini-Mental State Examination

The Mini-Mental State Examination (MMSE) is a brief cognitive screening instrument assessing global cognition (orientation, registration, attention, calculation, recall, and language) [42,43]. Scores range from 0 to 30 points, with scores of 23 or lower indicating cognitive impairment [42,43]. The MMSE is widely used clinically and in research to screen for cognitive impairment in older adults [43].

### 4.4. Emotional Screening Measures

#### 4.4.1. Beck Depression Inventory-II

The Beck Depression Inventory-II (BDI-II) is a 21-item self-report questionnaire assessing symptoms of depression in adults and adolescents [44]. Questions ask about cognitive and somatic depression symptoms, including symptoms such as pessimism, sadness, guilt, loss of pleasure, and suicidality [44]. Response options range from 0 to 3, with 0 indicating no experience of the specified symptom, and 3 indicating severe symptoms. A higher score indicates greater severity of depression symptoms, and scores greater than 13 indicate mild or greater depression symptoms. The BDI-II has been validated for use across many age groups, including older adults up to age 90 [44,45].

#### 4.4.2. General Anxiety Disorder-7

The General Anxiety Disorder-7 (GAD-7) questionnaire is a 7-item self-report assessment that screens for symptoms associated with generalized anxiety disorder, including nervousness, anxiety, worry, trouble relaxing, and irritability [46]. The GAD-7 assesses these symptoms over the last two weeks, and response options range from 0 to 3, with 0 indicating that symptoms were experienced “Not at all” and 3 indicating that symptoms were experienced “Nearly every day”, and scores greater than 4 indicating mild or greater anxiety symptoms [46]. The GAD-7 has been validated for use in older adults up to age 95 [46,47].

### 4.5. Statistical Analysis

All data were assessed for normality in accordance with the criteria proposed by Kline (2016) [48] and were found to be within acceptable limits. Preliminary correlations between age, education, BDI-II scores, GAD-7 scores, and cognitive ratings were conducted to identify potential covariates to control for in our analyses. Because BDI-II scores and GAD-7 scores were found to correlate with CFI scores, the associations between sleep measures, CFI scores, and global cognitive functioning were assessed using Pearson’s partial correlations while controlling for depression and anxiety scores (BDI-II and GAD-7). Age and education did not correlate with CFI scores in the preliminary correlations; however, because age is known to be associated with changes in sleep and cognition, age was included as a covariate, while education was not included as a covariate in partial correlations. Exploratory Pearson correlations were conducted to examine associations between PSQI component scores with CFI scores and global cognition. For correlations, significance was interpreted at *p* < 0.05, and correlation coefficients were interpreted as small (*r* = 0.10), medium (*r* = 0.30), and large (*r ≥* 0.50) [49]. All statistical analyses were performed using IBM SPSS Statistics version 30.0.

## 5. Conclusions

The current study revealed that greater subjective sleep disturbances, but not objective sleep disturbances, are associated with greater self-reported cognitive changes in older adults. While this study cannot determine the directionality of this association, improving sleep quality may reduce self-reported cognitive decline with age. Subjective sleep scales such as the ISI and PSQI could be used clinically to identify patients who may be at risk for concurrent subjective cognitive changes. The use of these sleep scales may also help identify patients warranting referral for objective sleep monitoring, such as polysomnography (e.g., those scoring >7 on the ISI or >5 on the PSQI). In tandem with sleep screening measures, clinicians should remain vigilant for complaints of subjective cognitive changes that may arise spontaneously during regular medical assessments, signaling that they should assess for and monitor concurrent sleep problems. Future research could expand on our findings by incorporating a more diverse sample, including more participants from different racial and ethnic groups, participants reporting greater subjective cognitive concerns, and participants reporting a wider range of mood symptoms. Additionally, future studies could examine other factors that might influence sleep, including retirement status, physical activity, and light exposure; they could also explore possible relations between sleep changes and neuroimaging and/or blood biomarkers associated with AD. Though further research is needed, these results add to our understanding of the relations between cognitive changes and sleep problems, both of which may be important clinical targets for preserving cognitive health in older adults.

## Figures and Tables

**Table 1:** Participant Characteristics. *n* = 45 (19 men, 26 women).

	**Mean**	**Standard Deviation**	**Range**
Age	64.2	9.5	52–85
Education	16.1	2.5	12–20
MMSE	28.2	1.5	24–30
CFI	2.1	2.4	0–9.0
PSQI	6.0	3.9	0–17
ISI	7.0	5.9	0–21
BDI-II	4.2	4.9	0–16
GAD-7	1.8	2.3	0–7

Note. MMSE: Mini-Mental State Examination; CFI: Cognitive Function Instrument; PSQI: Pittsburgh Sleep Quality Index; ISI: Insomnia Severity Index; BDI-II: Beck Depression Inventory-II; GAD-7: Generalized Anxiety Disorder 7-item.

**Table 2:** Pearson Correlations Between Scores on Cognitive and Subjective Sleep Measures.

	CFI			MMSE		
	*r*	[95% CI]	*p*	*r*	[95% CI]	*p*
Age	0.29	[−0.01, 0.54]	0.06	−0.05	[−0.34, 0.25]	0.76
Sex	0.03	[−0.26, 0.32]	0.83	0.01	[−0.28, 0.30]	0.94
Education	−0.07	[−0.36, 0.23]	0.63	0.22	[−0.08, 0.48]	0.14
BDI	**0.62**	**[0.40, 0.78]**	**<0.001** *	−0.13	[−0.41, 0.17]	0.38
GAD7	**0.31**	**[0.02, 0.56]**	**0.04** *	−0.05	[−0.34, 0.25]	0.76
ISI	**0.50**	**[0.25, 0.70]**	**<0.001** *	−0.04	[−0.39, 0.31]	0.79
PSQI	0.28	[−0.06, 0.57]	0.07	−0.24	[−0.54, 0.08]	0.12
Subjective Sleep Quality	**0.31**	**[0.02, 0.53]**	**0.048** *	−0.23	[−0.55, 0.11]	0.15
Sleep Latency	0.11	[−0.25, 0.46]	0.49	−0.09	[−0.37, 0.20]	0.59
Sleep Duration	**0.32**	**[0.03, 0.58]**	**0.04** *	−0.26	[−0.64, 0.13]	0.10
Sleep Efficiency	0.19	[−0.21, 0.53]	0.24	−0.11	[−0.41, 0.18]	0.49
Sleep Disturbance	0.13	[−0.18, 0.39]	0.43	−0.16	[−0.48, 0.15]	0.31
Sleeping Medication	**0.31**	**[0.02, 0.53]**	**0.048** *	−0.23	[−0.55, 0.11]	0.15
Daytime Dysfunction	−0.20	[−0.55, 0.17]	0.20	−0.02	[−0.23, 0.18]	0.93

Note. CFI: Cognitive Function Instrument; MMSE: Mini-Mental State Examination; ISI: Insomnia Severity Index; PSQI: Pittsburgh Sleep Quality Index. Statistically significant values are shown in bold and denoted with asterisks (*).

**Table 3:** Pearson Correlations Between Scores on Cognitive Measures and Objective Sleep Measures.

	CFI			MMSE		
	*r*	[95% CI]	*p*	*r*	[95% CI]	*p*
Sleep Onset Latency	0.14	[−0.27, 0.41]	0.40	−0.05	[−0.38, 0.25]	0.75
Total Sleep Time	0.18	[−0.08, 0.43]	0.28	0.26	[−0.05, 0.56]	0.12
Sleep Efficiency	0.07	[−0.23, 0.40]	0.70	0.12	[−0.15, 0.41]	0.47
Wake After Sleep Onset	−0.08	[−0.44, 0.29]	0.65	−0.08	[−0.39, 0.19]	0.64
Number of Awakenings	−0.06	[−0.28, 0.34]	0.73	0.10	[−0.24, 0.38]	0.54
Sleep Fragmentation Index	−0.03	[−0.37, 0.28]	0.86	−0.17	[−0.45, 0.10]	0.32

Note. *n* = 41 for actigraphy variables. CFI: Cognitive Function Instrument; MMSE: Mini-Mental State Examination.

## Data Availability

De-identified datasets are available upon reasonable request from the corresponding author (J.F.D.) and are subject to internal review and approval. Execution of a materials transfer agreement is required by our institution for transfer of data.
